# Is Blockchain Technology Suitable for Managing Personal Health Records? Mixed-Methods Study to Test Feasibility

**DOI:** 10.2196/12533

**Published:** 2019-02-08

**Authors:** Yu Rang Park, Eunsol Lee, Wonjun Na, Sungjun Park, Yura Lee, Jae-Ho Lee

**Affiliations:** 1 Department of Biomedical Systems Informatics Yonsei University College of Medicine Seoul Republic of Korea; 2 Clinical Research Center Asan Institute of Life Sciences Asan Medical Center, University of Ulsan College of Medicine Seoul Republic of Korea; 3 Department of Biomedical Informatics Asan Medical Center Seoul Republic of Korea; 4 Department of Emergency Medicine Asan Medical Center University of Ulsan College of Medicine Seoul Republic of Korea

**Keywords:** personal health record, mobile health, blockchain, Ethereum

## Abstract

**Background:**

There are many perspectives on the advantages of introducing blockchain in the medical field, but there are no published feasibility studies regarding the storage, propagation, and management of personal health records (PHRs) using blockchain technology.

**Objective:**

The purpose of this study was to investigate the usefulness of blockchains in the medical field in relation to transactions with and propagation of PHRs in a private blockchain.

**Methods:**

We constructed a private blockchain network using Ethereum version 1.8.4 and conducted verification using the de-identified PHRs of 300 patients. The private blockchain network consisted of one hospital node and 300 patient nodes. In order to verify the effectiveness of blockchain-based PHR management, PHRs at a time were loaded in a transaction between the hospital and patient nodes and propagated to the whole network. We obtained and analyzed the time and gas required for data transaction and propagation on the blockchain network. For reproducibility, these processes were repeated 100 times.

**Results:**

Of 300 patient records, 74 (24.7%) were not loaded in the private blockchain due to the data block size of the transaction block. The remaining 226 individual health records were classified into groups A (80 patients with outpatient visit data less than 1 year old), B (84 patients with outpatient data from between 1 and 3 years before data collection), and C (62 patients with outpatient data 3 to 5 years old). With respect to mean transaction time in the blockchain, C (128.7 seconds) had the shortest time, followed by A (132.2 seconds) and then B (159.0 seconds). The mean propagation times for groups A, B, and C were 1494.2 seconds, 2138.9 seconds, and 4111.4 seconds, respectively; mean file sizes were 5.6 KB, 18.6 KB, and 45.38 KB, respectively. The mean gas consumption values were 1,900,767; 4,224,341; and 4,112,784 for groups A, B, and C, respectively.

**Conclusions:**

This study confirms that it is possible to exchange PHR data in a private blockchain network. However, to develop a blockchain-based PHR platform that can be used in practice, many improvements are required, including reductions in data size, improved personal information protection, and reduced operating costs.

## Introduction

The ubiquity of mobile phones and rapid spread of wearable devices have greatly increased the amount and accuracy of data directly generated by patients outside of medical facilities [1-3]. These types of data, which are increasingly contributing to the use of personal health records (PHRs), are starting to attract as much attention as the patient data generated by medical institutions. Such data can be linked with the data collected at medical institutions, but it is expected that PHR management will become more personalized. This hope is one of the biggest drivers of change in the medical care paradigm, which is shifting its focus from general, guideline-based treatment to personalized treatment and disease prevention [3-8]. Although there are some advantages to this shift, there are economic, technological, regulatory, and sentimental barriers to the widespread adoption of PHR [9-11].

Recently, several studies have proposed the use of blockchain technology as a potential way to improve current PHR systems, which restrict access to and recording and sharing of data [12-16]. Blockchain databases and platforms are decentralized and irreversible; their advantages include reliability, transparency, and security [17,18]. Blockchain-based platforms are gradually expanding into a range of fields such as administration, insurance, and copyright act [19,20]. Distributed ledger technology (DLT) is the foundation of blockchain. DLT offers a consensus validation mechanism through a network of computers that facilitates peer-to-peer transactions without the need for an intermediary or centralized authority to update and maintain the information generated by the transactions. Each transaction is validated; a group of validated transaction is added and connected as a new “block” to an already existing chain of transactions, giving rise to the term “blockchain” [21]. In the medical field, the use of blockchain in electronic health records (EHRs), clinical trials, and drug tracking has been being proposed [22-24]. In clinical trials and research, there are several ways in which blockchain technology can improve the quality and processing of data [24]. For example, the smart contract function of the blockchain could be used to obtain consent from participants in a trial or track specific clinical trial events to improve the quality of a study [25]. Blockchain-based PHRs have been shown to solve technical and economic problems [12-14]. For example, instead of relying on a trusted third party, individuals may be able to manage their own data and can be assured of trust in the data through the blockchain. For example, after patients receive their medical records from medical institutions, these could be sent to other medical institutions, insurance companies, and research institutes, with the information being verified by the blockchain instead of a trusted third party. The medical information stored in patients’ mobile phones can be used without the help of a medical institution or a company. In addition, individuals can hand over their own data to data utilization parties, receive compensation for the data, and record the transaction details in the blockchain. To our knowledge, no published studies have examined the feasibility, effectiveness, performance, or costs of blockchain-based PHRs. The main questions that this study aimed to address were as follows: (1) Is the blockchain network suitable for PHR management? (2) How long does it take to share and distribute clinical data on a blockchain network? (3) How much does it cost to transmit clinical data in a blockchain network?

## Methods

### Study Design

To evaluate the usefulness of blockchain technology for the management of PHRs, we constructed a private blockchain network and conducted verification using real patient data. The blockchain-based PHR-sharing experiment was conducted on a 64-core, 398 GB Linux CentOS 6.9 server. The blockchain network was an Ethereum version 1.8.4–based private network [26], and 301 nodes were created from one local node via the Linux screen. We connected the additional 300 nodes to one main node representing a hospital.

To investigate the effectiveness of blockchain-based PHR management, we analyzed the time taken for data transactions on the blockchain nodes and the time taken for the spread of the clinical data over the network. Since the size of the clinical data is an important variable, we used data from 100 patients for each data size assessment. All clinical data were encoded with hexadecimal codes, and the transactions were performed with hex code in the transaction data field. In the same environment, 301 nodes were created, and one of them was assumed to be a hospital. The transactions for the clinical data of 300 patients were generated from the hospital node.

The times and costs associated with propagating the transactions of the 300 patient nodes were calculated. In order to calculate the propagation time for one transaction to all nodes in the network, the time until confirmation of the block containing the last transaction was defined as one cycle. The performance of each group was measured using the time required for one cycle and a measure of the propagation speed of the blockchain network according to the amount of data transmitted. In this private blockchain network, sending clinical data from a hospital node to patient nodes was repeated 100 times, and the time and cost were calculated as the average of these 100 iterations.

Cost was calculated using the gas fee, which is a special unit used in Ethereum networks. In Ethereum networks, every operation that can be performed by a transaction or contract costs a certain amount of gas, with operations that require more computational resources costing more gas than operations that require fewer computational resources [26]. For example, a high gas fee is engendered by a costly computation or an increase in the amount of data that must be stored in the node’s state. The gas fee is calculated by repeating 100 cycles of sharing and propagating clinical data in a blockchain network, in the same way as was done for calculating time.

### Data Sources

The clinical data, which forms the input to the PHRs, were collected from 300 patients randomly selected from the anonymized data warehouse of the Asan Medical Center in Seoul, South Korea [27,28]. Inclusion criteria were patients who had at least one outpatient visit record since 2014 and received at least one diagnosis and at least one laboratory test. The purpose of this study was to evaluate the capacity of blockchain technology to manage the patients’ clinical data. The patients were grouped into group A (100 patients with less than 1 year of outpatient data), group B (100 patients whose outpatient data were from between 1 and 3 years before data collection), and group C (100 patients with 3 to 5 years of outpatient visit data). We excluded all patient records that exceeded the 64 KB limit for blockchain transactions. The American Society for Testing and Materials’ Continuity of Care Record, a PHR standard, was used to fit the format of each patient’s clinical data [29].

### Data Analysis

We used Python 3.6.4 (Python Software Foundation) to obtain and analyze data for transaction logs between nodes. The transaction logs contained each node-specific data load time, the total network spread time, and the cost per transaction. We used analyses of variance (ANOVA) to test for statistical significance in the differences between the 3 groups. All reported *P* values were 2-sided, and *P* values less than .05 were considered significant. All statistical analysis was performed using R version 3.5.0 (R Foundation for Statistical Computing).

### Ethical Considerations

This study was approved by the Asan Medical Center’s Institutional Review Board (No. 2018-0178). The Ethics Committee waived the need for informed consent, as all data used in this study were anonymized and anonymously managed at all stages, including during data cleaning and statistical analyses.

## Results

### Overall Characteristics

Of the 300 patient records, the clinical data of 74 patients exceeded the 64 KB limit for transaction records in a blockchain: 20 from group A; 16 from group B; and 38 from group C. This elimination process left 226 individual health records in the final analysis: 80 in group A, 84 in group B, and 62 in group C (Table 1). All variables except gender were significantly different between the 3 groups. The number of visits, problems, medication, results, and procedures was highest in group A and lowest in group C.

### Blockchain-Based Data Transaction and Propagation

**Figure 1:**
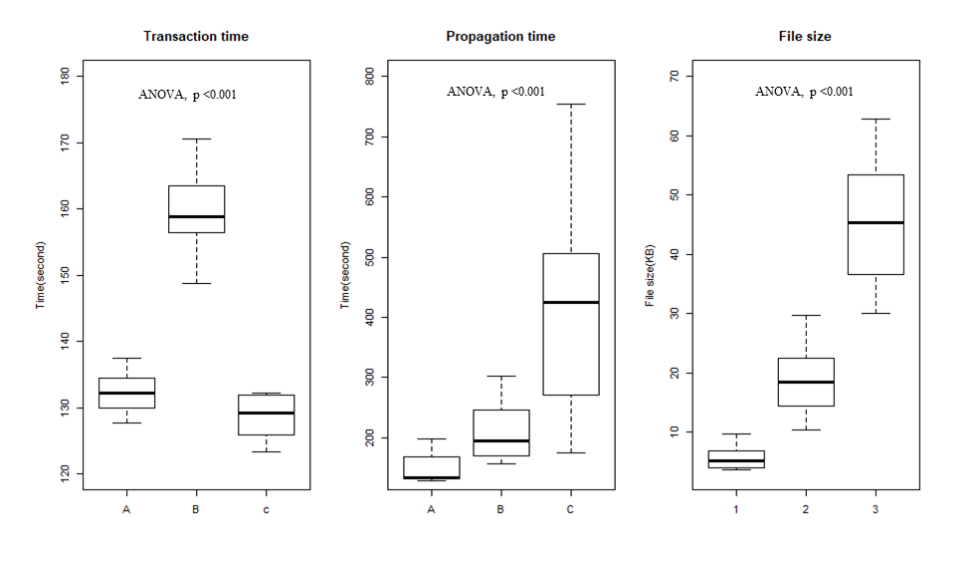
Mean transaction times, propagation times, and file sizes by group in a private blockchain network. The intergroup variance test for transaction time, propagation time, and file size was performed with analysis of variance and was found to be significant for all 3 variables.

Figure 1 shows comparisons of total transaction times and total propagation times among the 3 groups. The mean data sizes were 5.7 KB, 20 KB, and 37 KB for groups A, B, and C, respectively. In terms of mean transaction times, C (128.7 seconds) had the shortest time, followed by A (132.2 seconds) and B (159.0 seconds). The mean propagation times were 1494.2 seconds, 2138.9 seconds, and 4111.4 seconds for groups A, B, and C, respectively; the mean file sizes were 5.6 KB, 18.6 KB, and 45.38 KB, respectively.

**Table 1:** Basic characteristics of target groups.

Characteristics		Group A^a^ (n=80)	Group B^b^ (n=84)	Group C^c^ (n=62)
Gender, male, n (%)		37 (46)	32 (38)	32 (50)
Age, mean (SD)		44.27 (20.73)	45.67 (24.06)	55.89 (20.57)
**Diagnosis rank, n (%)**				
	1st (group A joint pain; groups B and C essential [primary] hypertension)	8 (10)	19 (23)	26 (42)
	2nd (group A essential [primary] hypertension; group B headache; group C chest pain, unspecified)	6 (8)	11 (13)	16 (26)
	3rd (group A cough; group B chest pain, unspecified; group C Encounter for gynecological examination)	6 (8)	9 (11)	13 (21)
Visit frequency, mean (SD)		4.58 (3.91)	17.21 (9.26)	32.70 (19.94)
Problems, mean (SD)		3.68 (3.79)	18.79 (10.29)	37.9 (25.30)
Medication, mean (SD)		3.35 (4.68)	31.71 (17.14)	74.4 (47.03)
Results, mean (SD)		3.38 (4.62)	33.09 (16.86)	75.59 (46.43)
Procedure, mean (SD)		0.04 (0.25)	1.34 (1.75)	2.57 (3.04)

^a^Less than 1 year of outpatient visit data.

^b^1 to 3 years of outpatient visit data.

^c^3 to 5 years of outpatient visit data.

### Blockchain-Based Data Operation Cost

To calculate gas fees, we divided the amount of gas required for each transaction in each group by the number of blocks actually executed. The gas consumed according to data size was found to be the same as the propagation speed. For a transaction to be propagated by each group, the blocks must be generated through a mining process. The mean number of blocks was 9.8 in group A, 9.5 in group B, and 17. 4 in group C. The larger the data size, the fewer the transactions included in one block.

**Figure 2:**
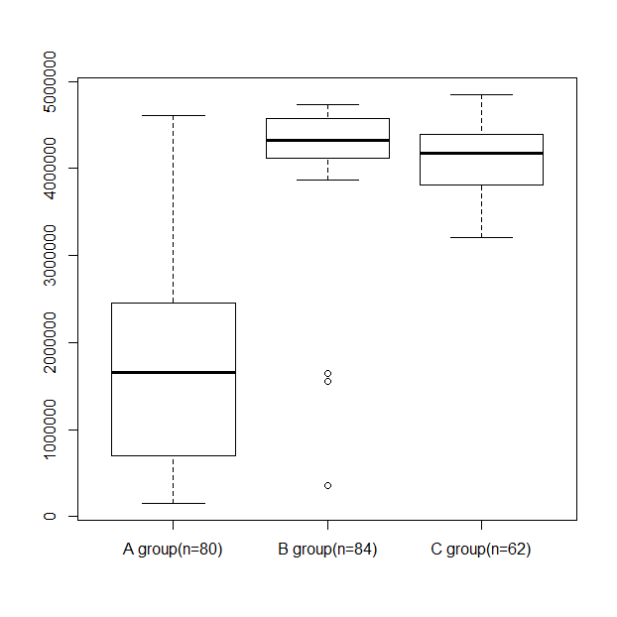
Mean gas consumption per group required to transact and propagate personal health records in a private blockchain network.

Figure 2 shows the mean gas consumption per group required for the transaction and propagation of PHRs in a private blockchain network: 1,900,767 for A; 4,224,341 for B; and 4,112,784 for C. The mean data size multiplied by the gas consumption showed the same trend for propagation of the gas cost. However, B and C had similar mean data sizes, indicating that the mean number of blocks in group C was much lower than that in groups A and B.

## Discussion

### Principal Findings

To the best of our knowledge, this is the first study to investigate the possibility of transferring data between hospital- and patient-based databases using real PHRs on a blockchain network. The limitations of sharing PHRs on a private Ethereum blockchain network include, first, the fact that sharing a PHR with a transaction in a blockchain cannot save more than 64 KB. This restriction in Ethereum is to prevent distributed denial-of-service (DDoS) attacks [26]. Because of this limit, data for 74 patients were excluded from this study. A DDoS attack is a method of attacking a Web server by causing abnormal traffic to flow from multiple computers to the Web server, causing the server to fail due to excessive traffic, process progress, and excessive input/output operations. DDoS attacks on blockchains can be accomplished by creating many large blocks with dust transactions. In order to prevent such attacks, the size of a transaction is limited. This limitation of the blockchain needs to be addressed to justify the use of blockchain as a PHR platform, since patient-generated health data (PGHD), socioeconomic data, and genomic data are becoming larger, as are the standard components of PHR [1,30,31]. Second, the larger the size of the data, the greater the gas consumption on the network, resulting in unnecessary operating costs. A pattern that we identified was that the basic cost of gas increases the cost of transactions at a higher rate with increasing amounts of data. In particular, PHRs may increase in data size over time, which may increase the operational costs associated with using a blockchain network. The costs of PGHD produced in real time via the Internet of Things or wearable devices will further increase over time [1,3,4]. Third, privacy and confidentiality considerations limit the current utility of blockchain for PHR management. In this study, data producers, owners, and content were all exposed. Exposure of a producer exposes the identity of the hospital and departments visited by a particular patient. The exposure of the transaction log means that the patient’s medical history is exposed; most people consider this to be sensitive information. Last, records cannot be erased once they are recorded. This is a fundamental characteristic of a blockchain, but in the case of medical data, the data may change or may need to be removed.

### Recommendations for Building a Blockchain Network for Personal Health Records

Transaction block size problems can be solved by choosing or designing blockchains that can accommodate high capacity. However, this modification could cause other problems, such as susceptibility to DDoS attacks [32], which cause data sizes to decrease. Therefore, there is a need for an alternative approach that can increase block data size without the associated security problems.

The time it takes to transact and propagate data using blockchain is a crucial variable in blockchain-based PHR. In this study, mean transaction time was longer with larger patient numbers, and mean propagation time was longer with larger file sizes but not with larger patient numbers. In this study, small patient sample groups were used for verification purposes. However, to allow for the management of and queries about large numbers of patients, it will be necessary to improve transaction and propagation times.

Increased operational costs due to large data sizes can be partially resolved by selecting a more advanced blockchain. The lack of privacy and confidentiality can also be improved by choosing a blockchain system that allows anonymization. The cryptographic technology called zk-SNARK (zero-knowledge succinct noninteractive argument of knowledge) is purported to allow users to hide both sender information and database content [33]. At present, however, anonymization techniques are imperfect and slow encryption speeds hinder performance.

Instead of writing all of the data, it is a good idea to record metadata such as data storage addresses, hash values, and timestamps in the blockchain. The actual data can then be stored elsewhere, such as on the hospital server, in patient mobile phones, or using a cloud-based storage system. This approach reduces the amount of data stored in the blockchain, regardless of the total database size, thereby freeing up storage space and minimizing costs. The approach also provides an alternative to storing personal and sensitive information on the blockchain and makes use of the advantages of both existing centralized storage technology and blockchain technology. At the same time, by allowing individuals to control storage, we can comply with the General Data Protection Regulation, which advises that “personal data shall be processed in a lawful manner, in a transparent manner in relation to the data subject” [34]. For blockchain to be optimized in a health care capacity, it should guarantee the right to health information for individuals that seek it. Recently, a model has been proposed that stores metadata in a blockchain and stores sensitive and large data in a separate storage such as a Cloud [35]. Until now, only the model has been presented, but we expect that services that implement these models will be released in the near future.

### Limitations of This Study

The main limitation of this study is that the feasibility evaluation of blockchain for PHR management was done on a private rather than a public network. However, this design choice was made due to legal limitations which prevented us from uploading sensitive personal data to public networks. In the future, research using public network–based blockchains, with participant consent, will be necessary.

To calculate network latency and throughput, it is necessary to install and operate the system on multiple computers instead of configuring several nodes on a small number of computers, as was done in this study. Future studies using multiple computers will be able to test feasibility by appropriately distributing physically separated computers having researchers, services, and the Cloud in other regions.

### Conclusions

Although many medical applications have been attempted using blockchain technology, studies investigating the feasibility and effectiveness of blockchain networks based on actual patient data have progressed relatively slowly. Our findings support the possibility of using blockchain technology to exchange actual patient data on a private blockchain network. Managing medical data using blockchain requires consideration of data size, operating costs, and privacy.

## Abbreviations

ANOVA: analysis of variance
DDoS: distributed denial of service
DLT: distributed ledger technology
EHR: electronic health record
PGHD: patient-generated health data
PHR: personal health record
zk-SNARK: zero-knowledge succinct noninteractive argument of knowledge

## References

[1] Kim J, Kam HJ, Park YR, Yoo S, Oh JS, Kim Y, Lee J (2018). Enchanted life space: adding value to smart health by integrating human desires. Healthc Inform Res.

[2] Walker DM, Sieck CJ, Menser T, Huerta TR, Scheck MA (2017). Information technology to support patient engagement: where do we stand and where can we go?. J Am Med Inform Assoc.

[3] Torkamani A, Andersen KG, Steinhubl SR, Topol EJ (2017). High-definition medicine. Cell.

[4] Sawesi S, Rashrash M, Phalakornkule K, Carpenter JS, Jones JF (2016). The impact of information technology on patient engagement and health behavior change: a systematic review of the literature. JMIR Med Inform.

[5] Singh K, Drouin K, Newmark LP, Filkins M, Silvers E, Bain PA, Zulman DM, Lee J, Rozenblum R, Pabo E, Landman A, Klinger EV, Bates DW (2016). Patient-facing mobile apps to treat high-need, high-cost populations: a scoping review. JMIR Mhealth Uhealth.

[6] Taki S, Lymer S, Russell CG, Campbell K, Laws R, Ong K, Elliott R, Denney-Wilson E (2017). Assessing user engagement of an mhealth intervention: development and implementation of the growing healthy app engagement index. JMIR Mhealth Uhealth.

[7] Sosa A, Heineman N, Thomas K, Tang K, Feinstein M, Martin MY, Sumer B, Schwartz DL (2017). Improving patient health engagement with mobile texting: a pilot study in the head and neck postoperative setting. Head Neck.

[8] Park YR, Lee Y, Kim JY, Kim J, Kim HR, Kim Y, Kim WS, Lee J (2018). Managing patient-generated health data through mobile personal health records: analysis of usage data. JMIR Mhealth Uhealth.

[9] Butler J, Carter M, Hayden C, Gibson B, Weir C, Snow L (2013). Understanding adoption of a personal health record in rural health care clinics: revealing barriers and facilitators of adoption including attributions about potential patient portal users and self-reported characteristics of early adopting users.

[10] Tang P, Ash J, Bates D, Overhage J, Sands D (2006). Personal health records: definitions, benefits, and strategies for overcoming barriers to adoption. J Am Med Informat Assoc.

[11] Lober W, Zierler B, Herbaugh A, Shinstrom S, Stolyar A (2006). Barriers to the use of a personal health record by an elderly population.

[12] Stagnaro C (2017). White paper: innovative blockchain uses in health care.

[13] Ivan D (2016). Moving toward a blockchain-based method for the secure storage of patient records.

[14] (2017). MediBloc: blockchain-based healthcare information ecosystem.

[15] Roehrs A, da Costa CA, da Rosa Righi R (2017). OmniPHR: a distributed architecture model to integrate personal health records. J Biomed Inform.

[16] Amar D, Choudhury O (2018). Blockchain for secure patient centered data capture and sharing.

[17] Nakamoto S (2008). Bitcoin: a peer-to-peer electronic cash system.

[18] Buterin V (2014). Ethereum: a next-generation cryptocurrency and decentralized application platform.

[19] Underwood S (2016). Blockchain beyond bitcoin. Communications ACM.

[20] Cohn A, West T, Parker C (2017). Smart after all: blockchain, smart contracts, parametric insurance, and smart energy grids. Georgetown Law Technol Rev.

[21] (2018). Blockchain technology and regulatory investigations.

[22] Wang H, Song Y (2018). Secure cloud-based EHR system using attribute-based cryptosystem and blockchain. J Med Syst.

[23] Mackey TK, Nayyar G (2017). A review of existing and emerging digital technologies to combat the global trade in fake medicines. Expert Opin Drug Saf.

[24] Benchoufi M, Ravaud P (2017). Blockchain technology for improving clinical research quality. Trials.

[25] Benchoufi M, Porcher R, Ravaud P (2017). Blockchain protocols in clinical trials: transparency and traceability of consent. F1000Res.

[26] Wood G (2014). Ethereum: a secure decentralised generalised transaction ledger.

[27] Shin S, Park YR, Shin Y, Choi HJ, Park J, Lyu Y, Lee M, Choi C, Kim W, Lee JH (2015). A de-identification method for bilingual clinical texts of various note types. J Korean Med Sci.

[28] Shin S, Kim WS, Lee J (2014). Characteristics desired in clinical data warehouse for biomedical research. Healthc Inform Res.

[29] Smolij K, Dun K (2006). Patient health information management: searching for the right model. Perspect Health Inf Manag.

[30] Eisenstein M (2015). Big data: the power of petabytes. Nature.

[31] Blazquez D, Domenech J (2018). Big Data sources and methods for social and economic analyses. Technological Forecasting Soc Change.

[32] McDowell M (2004). Security tip (ST04-015): understanding denial-of-service attacks.

[33] Sasson E, Chiesa A, Garman C, Green M, Miers I, Tromer E (2014). Zerocash: decentralized anonymous payments from Bitcoin.

[34] Rumbold J, Pierscionek B (2017). The effect of the general data protection regulation on medical research. J Med Internet Res.

[35] Chen Y, Ding S, Xu Z, Zheng H, Yang S (2018). Blockchain-based medical records secure storage and medical service framework. J Med Syst.

